# RADS ALPHABET: news and tips for young and general radiologists

**DOI:** 10.1186/s13244-025-02154-8

**Published:** 2026-01-12

**Authors:** Roberto Cannella, Carolina Lanza, Giuseppe Pellegrino, Domenico Albano, Alessandra Bruno, Giuditta Chiti, Caterina Giannitto, Elisabetta Giannotti, Cristiano Michele Girlando, Francesca Grassi, Carmelo Messina, Rebecca Mura, Giuseppe Petralia, Arnaldo Stanzione, Federica Vernuccio, Fabio Zugni, Antonio Barile, Nicoletta Gandolfo, Gianpaolo Carrafiello, Serena Carriero

**Affiliations:** 1 Section of Radiology, Department of Biomedicine, Neuroscience and Advanced Diagnostics (BiND), University of Palermo, Palermo, Italy; 2 Department of Radiology and Interventional Radiology, Foundation IRCCS Ca’ Granda Ospedale Maggiore Policlinico di Milano, Milan, Italy; 3 Department of Oncology and Hemato-Oncology, Università degli Studi di Milano, Milano, Italy; 4 IRCCS Istituto Ortopedico Galeazzi, Milano, Italy; 5 Department of Biomedical, Surgical and Dental Sciences, Università degli Studi di Milano, Milano, Italy; 6 Department of Clinical, Special and Dental Sciences, University Politecnica delle Marche, Ancona, Italy; 7 Istituto Fiorentino Analisi, Firenze, Italy; 8 Department of Biomedical Sciences, Humanitas University, Milan, Italy; 9 Radiology Unit, IRCCS Humanitas Research Hospital, Milan, Italy; 10 Cambridge Breast Unit, Cambridge University Hospital NHS Foundation Trust, Cambridge, United Kingdom; 11 Division of Radiology, IEO European Institute of Oncology IRCCS, Milan, Italy; 12 Division of Radiology, Università degli Studi della Campania “Luigi Vanvitelli”, Naples, Italy; 13 U.O.C. Radiodiagnostica, ASST Centro Specialistico Ortopedico Traumatologico Gaetano Pini-CTO, Milan, Italy; 14 Department of Biomedical Sciences for Health, Università degli Studi di Milano, Milan, Italy; 15 Department of Medicine and Surgery (DiMeC), University of Parma, Parma, Italy; 16 Department of Advanced Biomedical Sciences, University of Naples Federico II, Naples, Italy; 17 Department of Biotechnological and Applied Clinical Sciences, University of L’Aquila, L’Aquila, Italy; 18 Diagnostic Imaging Department, Villa Scassi Hospital-ASL 3, Genoa, Italy

**Keywords:** RADS, Structured reporting, Guidelines, Tips and tricks, Young radiologists

## Abstract

**Abstract:**

Reporting and Data Systems (RADS) aim at standardizing imaging acquisition, interpretation, lexicon, and reporting standards in specific patient populations, facilitating the communication between radiologists and clinicians. While the adoption of RADS has been supported by several studies and guidelines, with some of them endorsed by the American College of Radiology, the clinical adoption of the RADS algorithm remains heterogeneous among general practice radiologists worldwide, being lower in non-academic and young radiologists. This article aims to provide an updated review, aimed at young and general radiologists, of the RADS alphabet, discussing the main applications and imaging criteria with tips for their correct use in clinical practice. The following RADS will be discussed: BI-RADS, Bone-RADS, C-RADS, CAD-RADS, LI-RADS, Lung-RADS, MET-RADS-P, MY-RADS, NI-RADS, Node-RADS, O-RADS, ONCO-RADS, PI-RADS, ST-RADS, TI-RADS, and VI-RADS.

**Critical relevance statement:**

A comprehensive guide aimed at young and general radiologists featuring all of the major RADS with the objective to foster their implementation in clinical practice, which could be beneficial in a further standardization of the medical reports and in the communication between radiologists and clinicians.

**Key Points:**

- RADS are outlined to enhance communication efficacy between radiologists and clinicians.
- Updated overview of RADS frameworks, detailing applications, imaging criteria, and advancements.
- RADS’ implementation remains a challenge, but can be addressed.

**Graphical Abstract:**

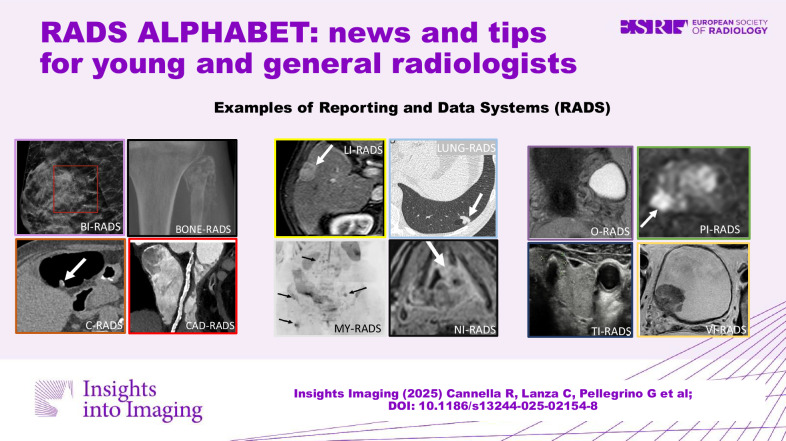

## Introduction

The Reporting and Data Systems (RADS) aim at standardizing imaging acquisition, interpretation, lexicon, and reporting standards in specific patient populations, facilitating the communication between radiologists and clinicians. RADS algorithms have been progressively developed and constantly updated by expert consensus committees, with many of them being progressively endorsed by the American College of Radiology and major society practice guidelines [1, 2]. Each algorithm provides comprehensive documents with tailored applications in each imaging modality (Table 1). The use of RADS in clinical practice has been supported by several studies providing evidence on the diagnostic performance and improvements for lesion detection and characterization compared to the non-standardized radiological interpretation. Nevertheless, adoption of the RADS algorithm remains heterogeneous among general practice radiologists worldwide, being lower in non-academic and young radiologists [3–5]. In our opinion, this is likely related to the complexity of the algorithms, lack of familiarity with the less commonly used systems, regional differences in the disease diagnosis and treatment, and constant updates of the systems over the past few years.

**Table 1:** Latest versions of RADS with their fields of application

Algorithm	Latest version	Technique	Application	ACR endorsement
BI-RADS				Yes
BI-RADS mammography, US, MRI	v2013	MMG, MRI, US	Malignancy risk assessment of breast pathology and management suggestions	
BI-RADS CEM	v2022	CEM	Standardized reporting for CEM findings, risk assessment and management suggestions	
Bone-RADS	v2023	XR	Malignancy risk assignment of bone lesions and management suggestions	Yes
C-RADS	v2023	CT	Screening/surveillance of colorectal cancer on CT-colonography	Yes
CAD-RADS	v2022	CT	Coronary artery disease on Coronary CT angiography	In collaboration
LI-RADS				Yes
LI-RADS US Surveillance	v2024	US	Surveillance ultrasound in high-risk patients	
LI-RADS CT/MRI	v2018	CT, MRI	Diagnosis of HCC in high-risk patients on CT/MRI	
LI-RADS CEUS	v2017	CEUS	Diagnosis of HCC in high-risk patients on CEUS	
LI-RADS CT/MRI Nonradiation TRA	v2024	CT, MRI	Response to nonradiation locoregional therapies on CT/MRI	
LI-RADS CT/MRI Radiation TRA	v2024	CT, MRI	Response to radiation locoregional therapies on CT/MRI	
LI-RADS CEUS Nonradiation TRA	v2024	CEUS	Response to nonradiation locoregional therapies on CEUS	
Lung-RADS	v2022	CT	Reporting and management of pulmonary findings on low-dose CT performed in lung cancer screening	Yes
MET-RADS-P	v2017	MRI	Metastatic disease evaluation and response assessment in advanced prostate cancer using whole-body MRI	No
MY-RADS	v2019	MRI	Disease detection and treatment response assessment in multiple myeloma using whole-body MRI	No
NI-RADS				Yes
NI-RADS ± PET	v2016	CT	CT surveillance with or without PET in patients with treated head and neck cancer	
NI-RADS MRI ± PET-CT	v2021	MRI, PET-CT	MRI with or without PET-CT in patients with treated head and neck cancer	
Node-RADS	v2021	CT, MRI	Assessment of lymph node involvement by cancer on CT/MRI	No
O-RADS				Yes
O-RADS US	v2022	US	Malignancy risk assessment of ovarian/adnexal lesions on US	
O-RADS MRI	v2022	MRI	Malignancy risk assessment of ovarian/adnexal lesions on MRI	
ONCO-RADS	v2021	MRI	Whole-body MRI for cancer screening	No
PI-RADS	v2.1 (2019)	MRI	Identify, localize and characterize findings in prostate MRI for treatment-naïve patients with suspected prostate cancer	Yes
ST-RADS	v2022	MRI	Differentiate between benign from malignant musculoskeletal soft tissue tumors of the extremities	No
ACR TI-RADS	v2017	US	Risk stratification system for thyroid nodules on US	Yes
EU-TI-RADS	v2017	US	Estimated risks of malignancy for thyroid nodules on US	No
VI-RADS	v2018	MRI	Diagnosis of muscle-invasive bladder cancer on MRI	In development

*CT* computed tomography, *MRI* magnetic resonance imaging, *MMG* mammography, *XR* X-ray plain film, *US* ultrasound, *CEUS* contrast-enhanced ultrasound, *CEM* contrast-enhanced mammography, *PET-CT* positron-emitting computed tomography

This article aims to provide an updated review, for young and general radiologists, of the RADS alphabet, discussing the main applications and imaging criteria with tips for their correct use in clinical practice.

## BI-RADS

The Breast Imaging-Reporting and Data System (BI-RADS) was the first published RADS system, and it has undergone significant evolution since its initial development in 1986 [6, 7]. BI-RADS is a standardized system used to categorize breast pathology seen on mammography, ultrasound (US), magnetic resonance imaging (MRI) and contrast-enhanced mammography (CEM). Its aim is to provide a standardized breast imaging report that includes a lexicon of descriptors, report organization, and a categorization system for audit and outcomes monitoring.

BI-RADS includes seven categories ranging from 0 to 6 [6, 7]. BI-RADS 0 indicates an incomplete study, necessitating additional imaging or previous mammograms for comparison [6, 7]. BI-RADS 1 signifies normal findings with no evidence of malignancy, and BI-RADS 2 indicates benign findings [6, 7]. Categories BI-RADS 3, 4, and 5 are defined by increasing probabilities of malignancy: BI-RADS 3 identify a finding with less than 2% chance of cancer; BI-RADS 4 is divided into three subcategories — 4A with low suspicion (2–9%), 4B with moderate suspicion (10–49%), and 4C with high suspicion (50–94%) of malignancy; BI-RADS 5 indicates findings highly suggestive of malignancy, with a probability greater than 95%. Finally, BI-RADS 6 is reserved for cases with known biopsy-proven malignancy [6, 7].

The release of the 5th edition in 2013 was a major milestone, offering standardized terminology and classification for breast imaging across various modalities [8]. BI-RADS continues to evolve, incorporating technological and scientific advancements. A key example is the 2022 release of the BI-RADS supplement for CEM [9]. Due to its higher sensitivity compared to digital mammography (95% vs 60%, respectively [10]), CEM has become a routine part of clinical practice in many breast imaging centers [10]. To accommodate this, a CEM-specific BI-RADS lexicon has been introduced, standardizing its application in clinical practice [11]. One of the most significant additions to this lexicon is the concept of “lesion conspicuity”, which describes the intensity of contrast enhancement relative to surrounding tissue. This feature has proven highly effective in predicting malignancy, especially in aggressive breast cancer types [12].

**Fig. 1:**
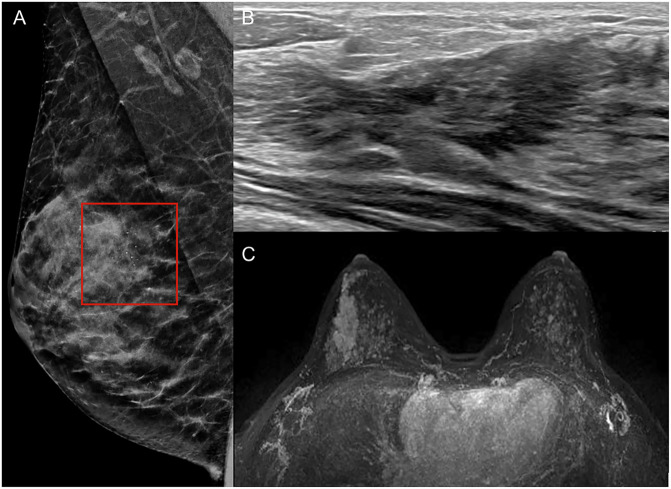
Fine pleomorphic regional calcifications (**A**, rectangle) observed in the upper outer quadrant of the right breast on mammography (BI-RADS 4a). **B** Corresponding hypoechoic non-mass with regional distribution is observed on US (BI-RADS 4a). **C** Segmental non-mass enhancement with heterogeneous enhancement is seen on MRI (BI-RADS 4a). Biopsy revealed fibroadenomatoid hyperplasia with non-specific granulomatous chronic inflammation (B2)

As BI-RADS continues to evolve, the upcoming 6th edition — currently in development — has already been previewed at major breast imaging conferences from 2023, revealing several important updates. For instance, in the mammography lexicon, descriptors like “lobular” for mass shape have been reinstated, while terms such as “punctate” calcifications, “milk of calcium”, and “popcorn-like” calcifications have been replaced with “coarse”, “layering”, and “round”, respectively. On US, new descriptors like the “echogenic rind” and categories for non-mass findings have been introduced (Fig. 1) [13, 14]. Similarly, on MRI, the removal of the term “focus” acknowledges its limited utility, as most small enhancements are benign [14–16], and it is now possible to differentiate mass from non-mass enhancement independently of size, due to increased spatial resolution of current techniques. Both MRI and US lexicons will now include classifications for normal and abnormal lymph nodes, with asymmetry in morphology or cortical thickening being considered suspicious, in certain clinical contexts. Another major change involves reclassifying certain “high-risk” histologies as “benign with upgrade potential”. Conditions such as lobular carcinoma in situ and atypical hyperplasia will now fall under BI-RADS 2, reflecting their biopsy-confirmed status [17].

BI-RADS’s role is diagnostic rather than prescriptive in the management of breast lesions. This point is crucial for a radiologist, and nowadays there are tools as Kaiser score in MRI, which is a diagnostic decision tool for distinguishing malignant from benign breast lesions [18]. This tool, by translating complex imaging findings and descriptors into a simple score, has a pivotal role in aiding radiologists in clinical decision-making. Looking to the future, it would be useful if tools like BI-RADS moved beyond their descriptive function to directly aid in decision-making.

The forthcoming 6th edition of BI-RADS is expected to further refine breast imaging reporting. By incorporating the latest technological advances and scientific insights, BI-RADS will continue to play a crucial role in improving the accuracy of breast cancer diagnosis and enhancing patient management across multiple imaging modalities.

Teaching point: BI-RADS provides probability-based categories (0–6) across imaging modalities (CEM, US, and MRI) to guide follow-up or biopsy of breast lesions.

## Bone-RADS

Several benign and malignant primary tumors may affect the skeleton, as well as secondary metastatic lesions [19]. Diagnostic uncertainty may arise due to different radiographic appearance and the wide spectrum of potential diagnoses [20, 21]. The Bone Reporting and Data System (Bone-RADS) scoring system was developed to standardize the evaluation and management of suspected neoplastic bone lesions using X-ray [22]. Of note, other structured approaches have also been proposed for incidental bone lesions detected on CT and MRI [23].

The Bone-RADS system evaluates different aspects of bony lesions to predict the risk of malignancy. These features are the margins as geographic with (IA) or without (IB) marginal sclerosis, ill-defined geographic margins (IC), non-geographic with moth-eaten (II) or permeative (III) osteolysis; the presence of periosteal reaction (aggressive or not); the endosteal erosion (from mild to deep); any pathological fracture; the extraosseous extension; the history of primary cancer [24–26]. A score is assigned to each of the above-described features, which increases according to aggressiveness. Additionally, the system includes the evaluation of matrix mineralization and tumor location to possibly predict histopathology [27, 28]. These are not considered in the final score.

**Fig. 2:**
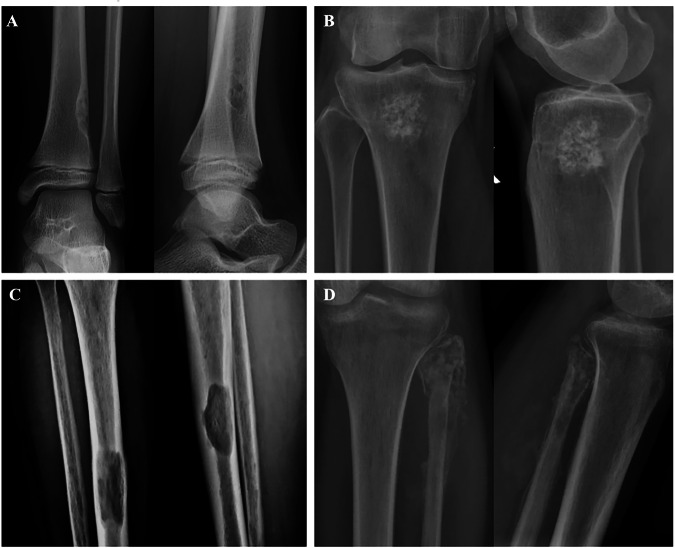
Four images showing the possible categories of Bone-RADS. **A** A non-aggressive eccentric lesion of the distal tibia (Bone-RADS 1), with the typical features of a non-ossifying fibroma. **B** A large enchondroma still with a non-aggressive pattern, but without clear marginal sclerosis and low risk of malignancy (Bone-RADS 2). **C** A well-defined lytic lesion in a subject with a previous history of renal cancer, which has certain aggressive features and possibly requires biopsy (Bone-RADS 3). **D** An extensively destructive lesion on the proximal fibula, with aggressive periosteal reaction and mass extending into the soft tissue (Bone-RADS 4)

Bone-RADS 0 is for incompletely characterized lesions, such as those affecting the axial skeleton, which can be poorly seen at radiography. Bone-RADS 1 are the classic “do not touch” lesions (e.g., non-ossifying fibroma, Fig. 2A), with very low risk of malignancy. Bone-RADS 2 are non-aggressive lesions without marginal sclerosis or causing endosteal scalloping, still with low risk of malignancy (e.g., enchondroma, Fig. 2B). Bone-RADS 3 are lesions at intermediate risk of malignancy, such as a well-defined lytic lesion in a subject with a previous history of cancer (Fig. 2C). Many of them will require biopsy. Bone-RADS 4 lesions are at high risk of malignancy and will be biopsied, such as osteolytic tumors with aggressive periosteal reaction and scalloping (Fig. 2D). In any case, symptoms should always be considered for the need for X-ray follow-up, as well as for the need for advanced imaging [23, 29, 30].

Teaching point: With the use of radiographic features like margin, periosteal reaction, and endosteal erosion, BONE-RADS stratifies bone lesions by malignancy risk (0–4).

## C-RADS

Initially published in 2005 [31] and then updated in 2023 [32], C-RADS has become the standard for reporting CT-colonography (CTC). C-RADS is based on the assessment of colonic and extracolonic findings. The assessment of colonic findings should be performed with an approximate window width of 1500 HU and an approximate window level of −200 HU [32]. With regard to colonic findings, a polyp is defined as a homogeneous soft tissue attenuation lesion projecting into the colonic lumen with a fixed point of attachment to the bowel wall. Polyps reported at CTC are typically 6 mm or larger, and their measurement should not include the stalk if present. For each polyp, four descriptors are assessed and used for categorization: lesion attenuation (i.e., soft tissue or fat containing), morphology (i.e., sessile, pedunculated, flat/laterally spreading, or mass of at least 30 mm), size (i.e., diminutive if 5 mm or less, 6–9 mm or at least 10 mm), and location (i.e., rectum, sigmoid colon, descending colon, transverse colon, ascending colon and cecum).

**Fig. 3:**
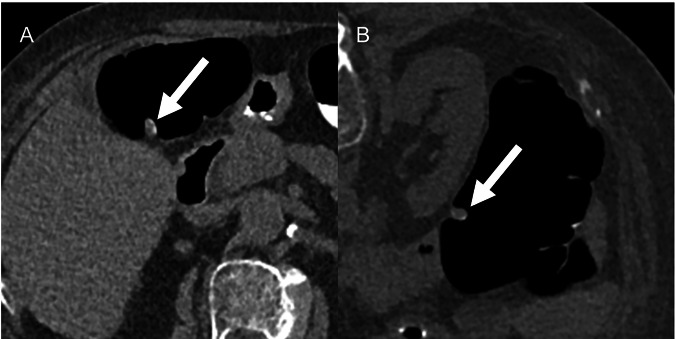
Supine (**A**) and prone (**B**) CT-colonography images in a 77-year-old female reveal a 8 mm polypoid lesion in the transverse colon. The lesion is classified as C-RADS C2a

Colonic and extracolonic findings are categorized from 0 to 4 (C0-C4 and E0-E4, respectively), as follows: the categories C0 and E0 are assigned in case of limited and inadequate CTC exam; the category C1 indicates a normal colon or presence of benign findings (e.g., colonic diverticula), and represent about 85% of the cases [33]; the category C2 includes polyps 6–9 mm in size and less than 3 in number (C2a) (Fig. 3), soft tissue mass or mass-like area that is likely benign, or stricture where malignancy cannot be entirely excluded (C2b); the category C3 includes polyp of at least 10 mm or at least 3 polyps, each 6–9 mm in size or polyps previously characterized as C2a that have enlarged at follow-up; the category C4 indicates a colonic mass that is likely malignant.

The categories E1 and E2 have been combined into a single E1/E2 category in the latest version of C-RADS and include normal CTC exams, likely benign unimportant findings or stable findings, and represent overall about 86.6% of the cases [33]; the category E3 indicates likely unimportant findings, incompletely characterized; the category E4 indicates likely important extracolonic findings, occurring in about 8.3% of cases, including unknown cancers in 1.5% [34].

According to C-RADS, based on the assigned categorization of colonic and extracolonic findings, routine screening every 5–10 years (i.e., for C1), CTC follow-up in 3 years (i.e., C2a lesions), colonoscopy referral (e.g., for C3), further work-up (e.g., for E4) or surgical/oncologic consultation (i.e., for C4) is indicated. Of note, although CTC follow-up in 3 years is usually recommended for C2a lesions, for some C2a lesions, referral to colonoscopy may be eventually indicated, depending on patient factors (e.g., age, comorbidities, preference), and local practice [32]. For C2b lesion, the management varies based on the clinical context and level of concern, and may range from CTC follow-up at 5 years in case of high likelihood of benignity to CTC follow-up at 3 years if benignity is less certain; if the clinical index of concern is high, there should be a category upgrade to C4, and flexible sigmoidoscopy or colonoscopy should be recommended [32].

Teaching point: C-RADS classifies colon and extracolonic CT-colonography findings into categories (C0–C4, E0–E4), streamlining screening intervals and colonoscopy referrals based on polyp size, number, and morphology.

## CAD-RADS

**Fig. 4:**
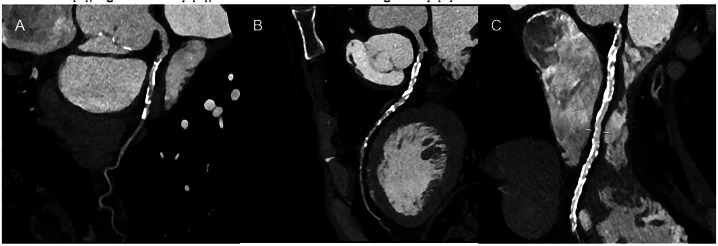
Trivasal coronary disease associated with CAD-RADS 5 classification (critical stenosis ≥ 70%) of circumflex (**A**), right coronary (**B**), and left descending artery (**C**)

Coronary Artery Disease Reporting and Data System (CAD-RADS) is a reporting framework that classifies findings of coronary CT angiography suggestive of coronary artery disease (CAD), with the aim of homogenizing and improving the communication between radiologists and clinicians [35, 36]. CAD-RADS classification yields information about the stenosis grade with a value ranging from 0 (no CAD) to 5 (severe CAD: ≥ 70% stenosis) while considering other factors such as high-risk anatomy and plaque morphology (Fig. 4). Modifiers are used to complement the classification and provide additional information (“N” for non-evaluable, “V” for high-risk plaque, “G” and “S” to signal the presence of bypass graft or stent, respectively) [37]. Since its first implementation in 2016, the clinical applicability has been confirmed by Maroules et al, where interobserver variability was excellent for both numerical value and modifiers (except for the V, which was deemed “fair”) [38]. In clinical application studies, it has been demonstrated that it predicts major adverse cardiovascular events with greater results than other risk stratification scores, such as a combination of Calcium Score and cardiovascular risk factors [39]. The system is currently limited because it does not fully evaluate the extent of the disease, considering only the most clinically relevant stenosis and the exact location of the stenosis. The stratified indication can be a double-edged sword and lead to mismanagement or mistreatment unless every patient is evaluated individually [40]. The renewed version of 2022 [41] adds to the standard classification system data about ischemic changes by CT fractional-flow-reserve (CT-FFR) or myocardial CT perfusion, enriching the model with more information about cardiac status. Ultimately, CAD-RADS enhances communication across multidisciplinary teams by offering a consistent and reproducible way of reporting results despite still having some pitfalls regarding specific clinical scenarios [42].

Teaching point: CAD-RADS grades coronary stenosis severity (0–5) on CT angiography and incorporates modifiers and CT perfusion/FFR data to guide cardiology management.

## LI-RADS

**Fig. 5:**
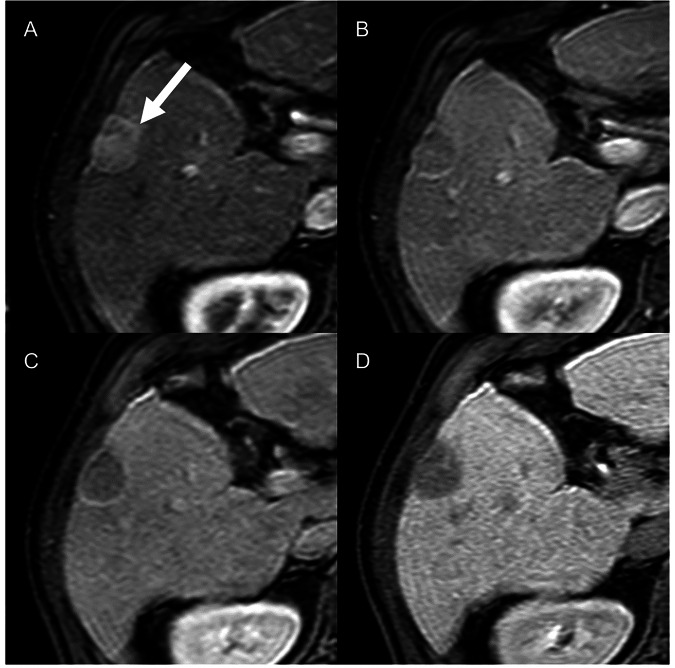
65-year-old male with hepatitis C-related cirrhosis. Gadoxetate disodium MRI demonstrates a 27 mm lesion in segment 5, with nonrim arterial phase hyperenhancement (**A**, arrow), nonperipheral washout and enhancing capsule on portal venous phase (**B**), hypointensity on transitional (**C**) and on hepatobiliary (**D**) phases as ancillary features favor malignancy. The lesion is categorized as LR-5 (definitively HCC)

The Liver Imaging Reporting and Data System (LI-RADS) has been designed to standardize the terminology, technique, interpretation, and reporting of liver lesions in patients at high risk of hepatocellular carcinoma (HCC) [43]. The LI-RADS algorithm includes an articulated collection of core documents aimed at ultrasound surveillance, diagnosis of HCC on cross-sectional imaging (CT, MRI, and CEUS), and evaluation of treatment response. Furthermore, the LI-RADS provides a comprehensive manual and a detailed standardized lexicon for imaging reporting [44, 45]. The latest CT/MRI LI-RADS v2018 has been integrated into the American Association for the Study of Liver Diseases practice guidance for the diagnosis of HCC [46]. Importantly, the LI-RADS diagnostic criteria can be applied only in high-risk patients, including patients with cirrhosis, chronic hepatitis B, even in the absence of cirrhosis, and current or prior history of HCC [47]. The LI-RADS diagnostic categories reflect the probability of an observation being HCC. The category is assigned using a stepwise algorithm and a diagnostic table combining the major features of HCC (size, nonrim arterial phase hyperenhancement, nonperipheral “washout,” enhancing “capsule,” and threshold growth) [48]. Additionally, ancillary features favoring malignancy or benignity can be applied at the radiologist’s discretion to adjust the final category [49]. The LR-5 category (definitively HCC) achieves a high specificity (> 90%) for the diagnosis of HCC (Fig. 5) [50, 51]. The LR-M category is used to define probably or definitively malignant observations without specific imaging features of HCC, although about 33% of them are proven to be atypical HCCs [52]. The LR-TIV (definitive tumor in vein) demonstrated a high specificity (99%) for the diagnosis of macrovascular invasion [53].

Recently, the LI-RADS treatment response algorithm has been updated with the release of two documents tailored to assessing response to nonradiation and radiation locoregional therapies, respectively [54, 55]. The key criteria to define a viable tumor have been redefined by the presence of mass-like enhancement in any post-contrast phase. The radiation algorithm introduces a new LR-TR nonprogressing category when the mass-like enhancement is stable or decreased in size after treatment [55]. Currently, LI-RADS cannot be applied to assess response to systemic treatments in patients with HCC.

Teaching point: LI-RADS categorizes liver observations (LR-1 to LR-5, LR-M, LR-TIV) in high-risk patients for HCC, combining major and ancillary imaging features to enhance specificity.

## Lung-RADS

Low-dose CT-based lung cancer screening allows for early detection of lung cancer, resulting in a reduction of specific mortality [56, 57]. In 2014, the American College of Radiology released the Lung CT Screening Reporting & Data System (Lung-RADS) to standardize reporting and management of lung cancer screening pulmonary nodules [58], with the last updated version being the Lung-RADS v2022 [59]. This includes categories from 0 (incomplete) to 4 (suspicious) — further subdivided into 4A, 4B, and 4X — and an S modifier for potentially significant findings unrelated to lung cancer [59]. Each category reflects a progressively increasing level of suspicion for malignancy and guides corresponding management recommendations [59].

Several nodule characteristics are evaluated for risk-categories assessment. Based on attenuation, pulmonary nodules can be distinguished in solid, non-solid and part-solid (with both ground-glass and soft tissue density) [60]. Regarding the lesion measurement, it is suggested to report the mean diameter to one decimal point, but v2022 also introduces volumetric measurement (mm^3^) [59, 61]. Nodule growth is currently defined as an increased mean diameter > 1.5 mm (or > 2 mm^3^) within a ≤ 12-month interval [59].

**Fig. 6:**
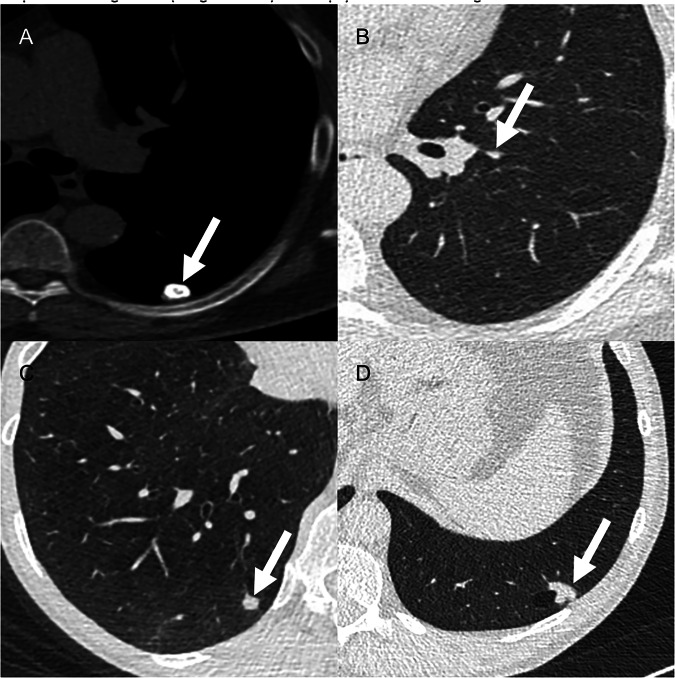
**A** Axial low-dose CT shows a solid nodule (arrow) in the left lower lobe with peripheral calcifications, consistent with pulmonary hamartoma (Lung-RADS 1). **B** Axial LDCT shows a perifissural solid nodule (arrow) in the left lower lobe, triangular in shape and measuring < 10 mm in the maximum diameter, likely representing an intrapulmonary lymph node (Lung-RADS 2). **C** Axial low-dose CT reveals a solid nodule (arrow) in the right lower lobe with a maximum diameter of 7 mm, categorized as Lung-RADS 3. **D** Axial low-dose CT shows a solid nodule (arrow) associated with a thin-walled cyst, measuring 18 mm in maximum diameter. Morphological features raised suspicion for lung cancer (Lung-RADS 4B), and biopsy confirmed the diagnosis of an adenocarcinoma

New classification criteria include atypical pulmonary cyst, since up to 22% of missed lung cancers at baseline were associated with cystic spaces [59, 62]. Airway nodules were already categorized as 4A, but v2022 provided additional guidance evaluating location, number, and persistence at follow-up [59]. The v2022 also extended the classification criteria of perifissural nodules, likely to represent intrapulmonary lymph nodes, to all juxtapleural nodules (Fig. 6) [59].

The newest version introduced the concept of “stepped management” with the timing of follow-up being based on the date of the current examination and not on baseline [59]. This approach is considered safe and limits frequent recall rates but also delayed follow-up intervals, like in 4A stable nodules, in which downgrading to Lung-RADS 2 could have been problematic considering the potential for slow-growing cancers [63–65]. Finally, v2022 recommends a 1 to 3-month follow-up low-dose CT for pulmonary findings suggesting an infectious/inflammatory process (S modifier) [59].

Teaching point: Lung-RADS standardizes nodule assessment in low-dose CT screening, using categories (0–4X) based on size, density, and growth to manage follow-up or referral for biopsy.

## MET-RADS-P

The Metastasis Reporting and Data System for Prostate Cancer (MET-RADS-P) guidelines were developed to standardize the acquisition, interpretation and reporting of whole-body MRI (WB-MRI) in patients with advanced prostate cancer, with the goal of facilitating its adoption in clinical trials [66]. WB-MRI showed substantially higher diagnostic performances compared to bone scintigraphy and CT, but prospective trials investigating its prognostic impact are still awaited [67–69]. The MET-RADS-P provided a core protocol and extensions for comprehensive assessment, covering from the vertex to mid thighs, including morphologic T1- and T2-weighted images, diffusion-weighted imaging (DWI) and mandatory reconstructions such as 3D maximum intensity projections of high *b*-value images, apparent diffusion coefficient (ADC) maps and fat image reconstructions [66]. Through standardized reporting, MET-RADS-P guidelines help promote consistent response assessment of metastatic disease across readers of different expertise [70, 71].

MET-RADS-P define a set of image interpretation criteria, both dimensional, such as lesion diameter, and functional, such as signal intensity in DWI and ADC value, which must be applied to identify active disease across 14 anatomic regions included in the standardized report: skeleton (7 regions), lymph nodes (3), visceral sites (3) and primary disease [72]. The extent of active disease identified following these criteria was associated with cancer-specific survival in initial retrospective reports [73].

When WB-MRI is performed after systemic treatment, five different patterns of response of metastases can be defined, termed Response Assessment Categories (RAC), ranging from RAC 1 (highly likely to be responding) to RAC 5 (highly likely to be progressing). These RACs must be assigned to each anatomic region affected by the disease; secondary RACs may be assigned to each anatomic region in the presence of heterogeneous response (Supplementary Fig. 1).

Teaching point: MET-RADS-P facilitates structured whole-body MRI assessment in advanced prostate cancer by scoring regional metastatic activity (RAC 1–5), supporting response evaluation.

## MY-RADS

MRI is established as the gold standard for skeletal assessment in multiple myeloma, and overcomes the limitations of CT in assessing treated disease [74–76]. International guidelines recommend WB-MRI as second-line imaging in suspected myeloma and as first line for the staging of solitary bone plasmacytoma [77]. The emerging role of WB-MRI led to the development of the Myeloma Response Assessment and Diagnosis System (MY-RADS), with the aim of standardizing the acquisition, interpretation and reporting of this imaging technique [78].

MY-RADS provides a core protocol for skeletal disease detection and a comprehensive assessment protocol for the evaluation of extramedullary disease and treatment response. The acquisition covers from vertex to knees, including morphologic T1- and T2-weighted images, DWI and mandatory reconstructions: 3D maximum intensity projections of high *b*-value images, ADC maps, fat and water images and fat fraction maps. Like MET-RADS-P, MY-RADS guidelines also promote a multiparametric evaluation of the bone marrow, by means of morphologic and functional image interpretation criteria, recording the extent of disease in a standardized report, comprising seven skeletal regions and extramedullary sites. In each region, the pattern of marrow infiltration can be classified as focal, micronodular, diffuse, focal on diffuse. Qualitative evaluation of the disease burden can be obtained through a numerical scoring system. This system, in combination with ADC and fat fraction measurements, was found to predict deep treatment response with 93.5% accuracy in a retrospective series of patients without anemia [79].

**Fig. 7:**
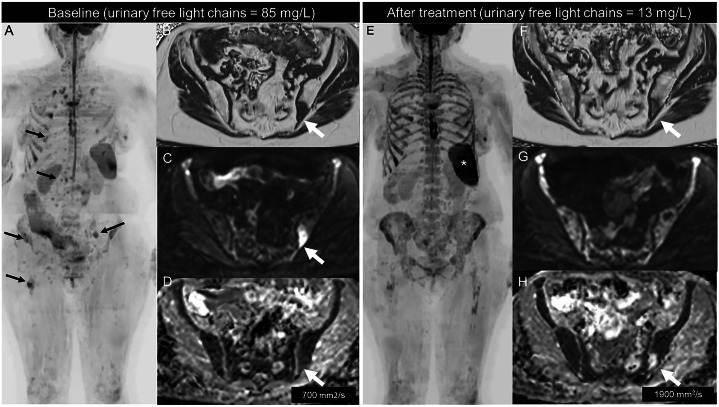
Whole-body MRI with MY-RADS protocol in a patient with relapsing light chain multiple myeloma, before (left) and after (right) treatment with Daratumumab, pomalidomide and dexamethasone and bone marrow stimulating factors. Bone involvement with a focal pattern can be noted in multiple skeletal regions in the coronal maximum intensity projection of high b-value DWI, displayed in inverted grayscale (arrows in **A**). Axial sections show a focal lesion in the left iliac bone (arrows) with low fat content in the fat fraction map (**B**), high signal intensity in high b-value DWI (**C**), and low ADC values (**D**). After treatment, diffuse intermediate intensity signal is visible on DWI images (**E**) across the whole skeleton, without corresponding bone marrow infiltration on fat fraction; spleen enlargement is also present (asterisk in **E**). Notably, this finding is related to the administration of bone marrow-stimulating factors and does not indicate disease progression. On the other hand, focal lesions such as the one in the left iliac bone show decreased signal in DWI and are not visible (**E**, **G**), initial fatty repopulation (arrow in **F**) and markedly increased ADC (arrow in **H**), findings suggesting high likelihood of response (RAC 1)

When serial WB-MRI is used for treatment monitoring, such as in nonsecretory, oligosecretory and extramedullary disease [80], five patterns of response (RAC) can be assigned, akin to those used in MET-RADS-P guidelines (Fig. 7), ranging from RAC 1 (highly likely to be responding) to RAC 5 (highly likely to be progressing). RAC 1 pattern was associated with superior overall survival in a recent retrospective study, also reporting high inter-reader agreement in the classification of response [81].

Teaching point: MY-RADS standardizes WB-MRI for multiple myeloma, using morphological and functional criteria to assign disease burden and response scores (RAC 1–5) in bone and soft tissue.

## NI-RADS

**Fig. 8:**
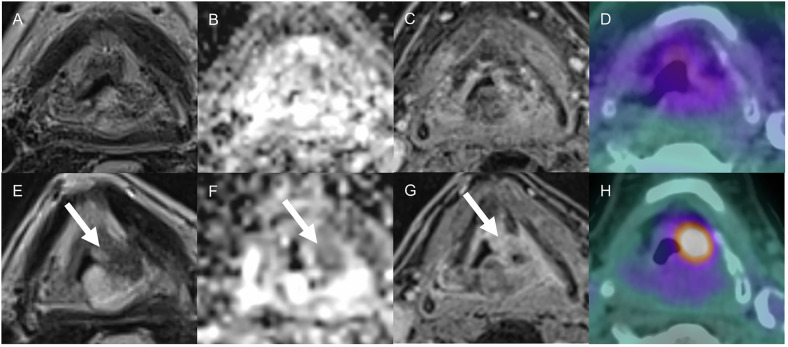
59-year-old man with a history of c4N1 oropharynx squamous cell carcinoma, positive for human papillomavirus after chemo and radiotherapy. Axial T2 TSE sequence (**A**), ADC map (**B**), T1 sequence after contrast injection (**C**), and FDG-PET/CT (**D**) showing negative follow-up examinations. MRI obtained 3 years post-treatment (**E**–**G**) demonstrates a nodule at the primary site (arrows) with intense focal FDG uptake (**H**). Primary site was assigned to the NI-RADS 3 category. Biopsy was performed and confirmed recurrence

Head and Neck Imaging Reporting and Data System (NI-RADS) was developed by the American College of Radiology to standardize the reporting of surveillance imaging with linked management recommendations in patients with treated head and neck cancer [82]. Based on contrast-enhanced CT, MRI, or Fluorodeoxyglucose-PET/CT findings and on comparison with baseline imaging, this system assigns a category from 1 to 4 according to the level of suspicion of recurrence at the primary site and regional lymph nodes [83–86]. These findings include nodular or ill-defined non-mass-like contrast enhancement on the primary site, changes in signal intensity on MRI with DWI with ADC map, perineural spread, residual or new enlarging nodes with their morphologic features, and FDG uptake. The examination is classified as NI-RADS 0 if the evaluation is currently incomplete due to unavailable prior imaging, which will be obtained for comparison. Category 1 represents no evidence of recurrence and recommends routine surveillance. Category 2 indicates low suspicion for recurrence at the primary site or node and suggests direct visual inspection for superficial abnormality, and short-term follow-up or PET/CT for deep abnormality or node suspicion. Category 3 suggests high suspicion for recurrence and recommends biopsy (Fig. 8). Category 4 represents histologically or clinical-radiologically definite recurrence.

Prior studies demonstrated strong or moderate inter-reader agreement for both the primary site and the neck using contrast-enhanced CT [87], moderate for the primary site and substantial for the neck using MRI, and almost perfect for the primary site using DWI-MRI [88]. According to a recent meta-analysis [89], NI-RADS demonstrated high specificity (94%) but moderate sensitivity (69%) in post-treatment imaging surveillance. The recurrence rate for NI-RADS categories 1–3 was 3%, 13%, and 77%, respectively. However, the diagnostic performance of NI-RADS may be influenced by factors such as the type of analysis (per patient vs. per site) and the number of sites evaluated.

Teaching point: NI-RADS guides post-treatment head and neck cancer surveillance via imaging categories (1–4) with linked clinical actions, balancing recurrence risk against biopsy and imaging follow-up.

## Node-RADS

Lymph node involvement is a significant characteristic in oncology that affects staging, prognosis and patient management, often distinguishing surgical candidates from non-surgical care [90, 91]. Previously, the main factor that used to support a suspicion of lymph node involvement was only the size of the lymph nodes [92], but size alone is actually thought to be a poor indication for predicting the lymph node localization of the disease, since a single cut-off size cannot be used for all body districts due to variations in the cut-off size values depending on the patient’s age and anatomical location.

Node-RADS 1.0 was introduced in 2021 to provide a standardized assessment of lymph nodes in cancer and to classify the degree of suspicion of lymph node involvement using a combination of criteria [93]. In addition to the traditional criterion of “size” (“normal”, “enlarged” and “bulk”), Node-RADS includes the criterion “configuration” with morphological categories of “texture”, “border” and “shape” for the evaluation of each individual lymph node. These two scoring criteria (size and configuration) are combined into rating categories ranging from 1 (“very low probability”) to 5 (“very high probability of cancer”) (Supplementary Fig. 2) [93].

Node-RADS can be applied to any anatomical site and to both regional and non-regional lymph nodes in relation to the location of a primary tumor being evaluated by CT and/or MRI. Node-RADS can be used in addition to the other RADS (such as LI-RADS or PI-RADS). An exception is NI-RADS in head and neck cancer surveillance, where a score is already assigned for cervical (“neck”) lymph nodes; node-RADS and NI-RADS do not agree on their criteria and algorithm [93, 94]. Several studies in the literature have shown that structured assessment of lymph nodes using Node-RADS improves diagnostic accuracy, facilitates uniformity of staging and evaluation of response to treatment [94–96].

Teaching point: Node-RADS uses a combined score of size and morphology (1–5) across all body regions to assess lymph node malignancy probability, aiding staging and treatment planning.

## O-RADS

The Ovarian-Adnexal Reporting and Data Systems (O-RADS) for US and MRI were developed by the American College of Radiology to standardize the lexicon, risk stratification, and management of ovarian and adnexal lesions. It supports radiologists in risk stratification, aiming to reduce unnecessary follow-up and overtreatment while expediting the evaluation of suspected ovarian cancer. Ultrasound is the first-line imaging modality, with a transvaginal approach, when feasible, and O-RADS stratifies adnexal lesions into six categories (O-RADS US 0–5). The updated O-RADS v2022 introduces new lexicon descriptors to improve diagnostic specificity for low-risk lesions, including the term “bilocular” for cystic lesions and “shadowing” for smooth solid lesions, and eliminates the previous “indeterminate” category, grouping lesions into risk categories: almost certainly benign, low risk, intermediate risk, and high risk for malignancy [97].

Most adnexal lesions detected on ultrasound can be accurately characterized as physiologic (e.g., a simple cyst or corpus luteum < 3 mm, O-RADS US 1) or almost certainly benign when classical benign imaging features are present and lesion size is < 10 cm, including hemorrhagic cysts, endometriomas, or dermoid cysts (O-RADS US 2, malignancy risk < 1%), for which conservative management is recommended [98–100].

When classic benign features are absent, the risk of malignancy ranges from 1% to > 50%, due to overlapping imaging characteristics and operator dependency. Based on morphological and vascular features, lesions are classified as O-RADS US 3 (low risk, 1–10%), O-RADS US 4 (intermediate risk, 10–50%), or O-RADS US 5 (high risk, ≥ 50%). Follow-up US, MRI, or referral to a gynecologic oncologist is recommended depending on the risk category.

MRI provides superior characterization of both fluid and solid components of adnexal lesions and is indicated when higher specificity is required, when a lesion is incompletely assessed by US, or when its origin is indeterminate [101]. MRI also plays a crucial role in preoperative evaluation when fertility-sparing surgery is considered [102].

**Fig. 9:**
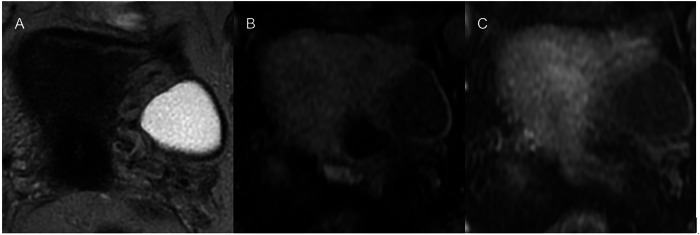
MRI in a 62-year-old patient following the detection of a cystic formation during transvaginal ultrasound. In the left adnex, an unilocular cyst measuring 34 mm with simple fluid content, hyperintense on T2 (**A**) and hypointense on T1 (**B**), with a thin wall and no enhancement on post-contrast image (**C**). Related to the dimensions and the postmenopausal status of the patient, the O-RADS MRI score 2 is assigned

The O-RADS MRI risk stratification system includes six categories: O-RADS MRI 0 (incomplete examination), O-RADS MRI 1 (normal ovaries), O-RADS MRI 2 (almost certainly benign; PPV < 0.5%), O-RADS MRI 3 (low risk; PPV ~ 5%), O-RADS MRI 4 (intermediate risk; PPV ~ 50%), and O-RADS MRI 5 (high risk; PPV ~ 90%), with an overall diagnostic accuracy of 92% [101] (Fig. 9).

MRI should be performed using a 1.5-T or 3-T scanner with a multiparametric protocol, including sequences tailored to characterize adnexal lesions. Dynamic contrast-enhanced (DCE) perfusion imaging is recommended for generating time–intensity curves (TICs) for the lesion and myometrium, facilitating risk stratification.

At MRI, any non-fluid part of an adnexal lesion is considered a solid component and is divided into two categories: solid tissue, which must enhance, and non-enhancing solid components, such as blood clots or avascular debris.

In the absence of solid tissue, the risk of malignancy approaches 0%. When the solid tissue appears hypointense relative to skeletal muscle on both T2-weighted and DWI sequences (“dark T2/dark DWI” pattern), the lesion is considered benign (O-RADS 2) regardless of enhancement characteristics.

If the “dark T2/dark DWI” pattern is absent, risk categorization is guided by TIC analysis of the solid tissue relative to the myometrium on DCE imaging. Type 3 TIC (rapid enhancement greater than the myometrium with plateau) indicates high malignancy risk (O-RADS MRI 5), type 2 TIC (moderate enhancement less than the myometrium with plateau) suggests intermediate risk (O-RADS MRI 4), and type 1 TIC (minimal gradual enhancement without plateau) is typical of benign lesions (O-RADS MRI 3).

In post-hysterectomy patients, TIC types 2 and 3 cannot be distinguished, and categories 4 and 5 are not differentiable. When DCE imaging is unavailable, stratification is based on relative enhancement at 30–40 seconds: O-RADS MRI 4 if less than myometrium, O-RADS MRI 5 if greater [102].

Ovarian and adnexal reporting presents unique challenges compared to other reporting lexicons due to physiological ovarian changes and differences between the two systems, reflecting the strengths and limitations of each imaging modality.

Key limitations include variability in malignancy risk estimates between modalities — most notably in O-RADS 4 (10–50% for US vs. ~ 50% for MRI) — and the classification of certain specific lesions into different categories in O-RADS US vs MRI. Furthermore, O-RADS MRI currently lacks management recommendations for each risk category, which are included in O-RADS US.

Teaching point: O-RADS integrates US and MRI risk stratification systems (scores 0–5) to optimize adnexal lesion triage, enhancing malignancy detection.

## ONCO-RADS

Whole-body MRI is currently recommended for cancer screening in both adult and pediatric patients with tumor predisposition syndromes, including Li-Fraumeni syndrome, hereditary paraganglioma and pheochromocytoma syndromes, constitutional mismatch repair deficiency syndrome and hereditary retinoblastoma [103–106]. In 2021, the ONCO-RADS (Oncologically Relevant Findings Reporting and Data System) guidelines were introduced, offering a standard protocol for WB-MRI image acquisition in these patients [107]. This protocol can be adapted with extensions to evaluate specific areas associated with individual syndromes or through the use of contrast media. Additionally, a “short protocol” can be used for cancer screening in asymptomatic individuals from the general population (Supplementary Fig. 3) [108].

ONCO-RADS provides a structured reporting template linked to a five-category classification system, distinguishing between benign findings, findings that require specific follow-up, and findings that are suspicious for neoplasm. This system is designed to improve communication of exam results between clinicians and patients and to ensure consistent reporting, which is crucial in annual serial screenings [107]. In a retrospective study of 2064 asymptomatic individuals, Hu et al demonstrated that the ONCO-RADS categories not only enhance communication but also support a risk-based management approach, confirming a positive correlation between the ONCO-RADS classification and cancer risk [109].

Teaching point: ONCO-RADS provides a structured WB-MRI framework for cancer screening in high-risk patients, assigning categories to enhance detection, consistency, and risk-tailored follow-up.

## PI-RADS

Prostate MRI has revolutionized the diagnostic pathway of prostate cancer, with the current paradigm being the “MRI-first” approach, which reduces unnecessary biopsies while allowing the detection of more clinically significant prostate cancer compared to systematic biopsy alone [110, 111]. This is at least partly due to the introduction of the Prostate Imaging Reporting and Data System (PI-RADS), whose adoption for prostate MRI acquisition and interpretation is strongly recommended by the European Association of Urology guidelines [112]. According to PI-RADS, prostate MRI should include high-resolution T2-weighted and DWI sequences (with ADC map and a separate high *b*-value set of images > 1400 s/mm^2^) with a dedicated field of view, as well as a DCE sequence [113]. The first two are considered dominant sequences, respectively, for the classification of transition and peripheral zones findings. Similar to other RADS, a five-point probability scale is proposed, with scores 1 and 5 representing the lowest and highest probability of clinically significant prostate cancer presence [113].

**Fig. 10:**
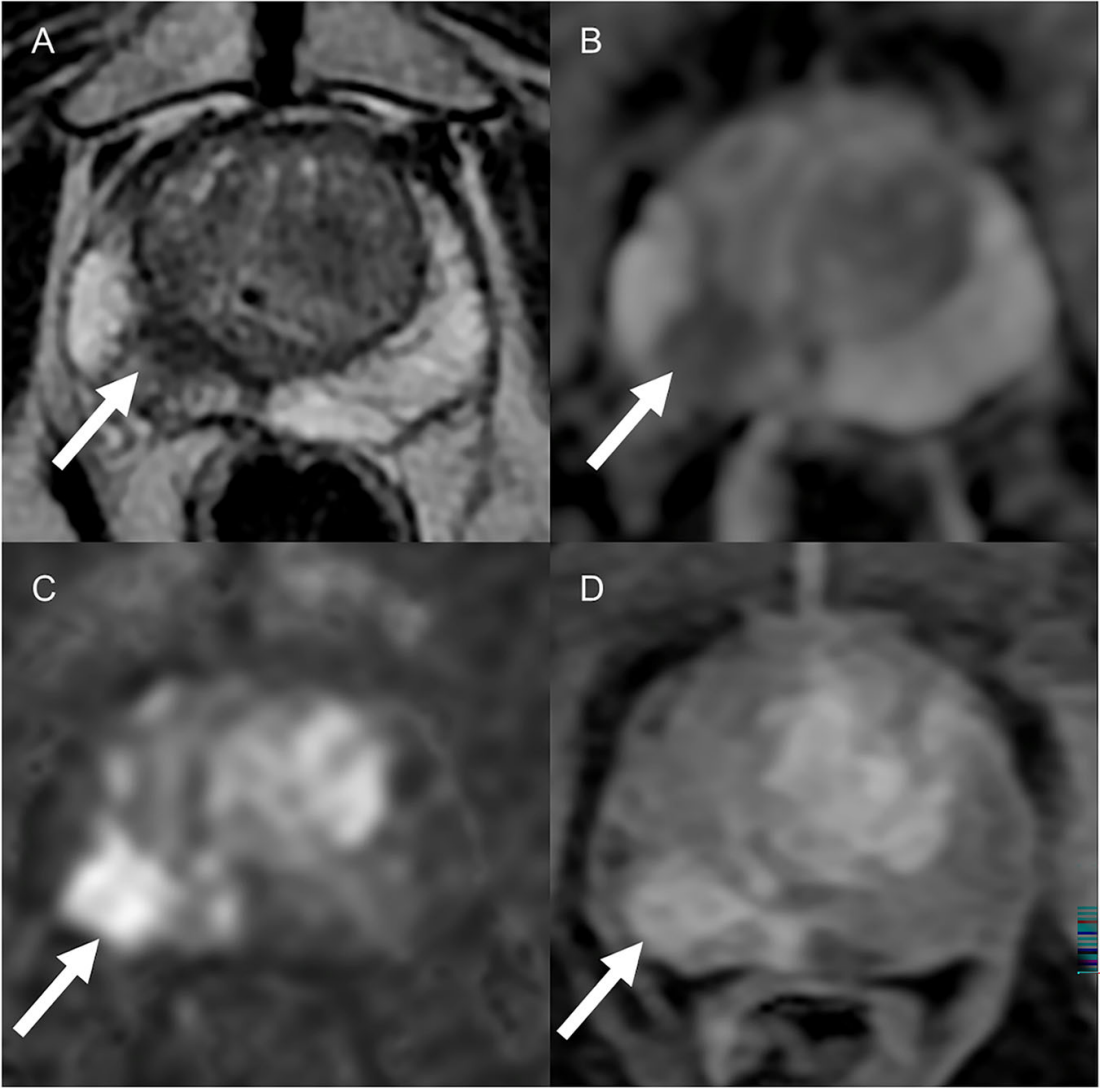
The T2-weighted axial image detects a lesion in the peripheral zone of the right lobe (**A**). The ADC (**B**) and DWI (**C**) maps demonstrate a focal area of markedly hypointense/hyperintense signal in the ADC map and high b-value, with corresponding positive DCE findings (**D**), consistent with PI-RADS 5 (arrows)

Focal areas in the peripheral zone showing markedly hypointense/hyperintense signals on ADC/high *b*-value DWI, respectively, are scored PI-RADS 4. When the greatest dimension is ≥ 15 mm, or in case of definite signs of extraprostatic extension, the same finding will be scored as PI-RADS 5 (Fig. 10). If signal changes on ADC/high *b*-value DWI are focal but only mild, a score of 3 (indeterminate) should be assigned, which can be upgraded to 4 in case of positive DCE findings (focal early or contemporaneous enhancement in the corresponding area). The transition zone, in which benign prostatic hyperplasia occurs, has a heterogeneous MRI appearance, with circumscribed and encapsulated nodules arranged in an “organized chaos.” In this context, suspicious findings in the transition zone are represented by lenticular or non-circumscribed areas of moderate and homogeneous hypointense signal in T2-weighted images (the same rules as seen in the peripheral zone are used to discriminate between PI-RADS 4 and 5 lesions). Radiologists should be aware of mimics and pitfalls in prostate MRI, and experience is needed to master the complexities of prostate MRI and PI-RADS [114–117].

Teaching point: PI-RADS scores prostate MRI lesions (1–5) based on DWI, T2, and DCE criteria, distinguishing clinically significant prostate cancer and guiding biopsy with high specificity.

## ST-RADS

Musculoskeletal soft tissue tumors are frequently found at the extremities, ranging from benign lipomas or cysts to more aggressive malignant sarcomas. While benign tumors are much more common, malignant tumors may be aggressive with significant morbidity, mortality and financial burden [118]. MRI is the modality of choice for tumor characterization, but diagnosis based on MRI features only remains challenging, and biopsy is frequently required for histopathological confirmation [21, 119, 120].

The Soft-tissue Tumor Reporting and Data System (ST-RADS) has been recently proposed to help radiologists in differentiating ST-tumors based on the WHO classification [121]. MRI categorization differentiates between adipocytic appearance, T2-hyperintense or hypointense lesions. Seven categories were identified, from ST-RADS 0 (incomplete imaging study) to ST-RADS VI (biopsy-proven malignant tumor). Of note, the use of DWI is considered supplemental utility and not included in the standard MRI protocol [121].

Clear features of benignity are the uniform fat signal with full suppression on saturated images, the lack of enhancement, dimension < 10 cm, and a fully calcified or fluid signal mass. The chance of malignancy increases with the proximity to joints, fasciae or bursae, the presence of septations, the increasing amount of T2-hypointense fibromatous tissue, and thin peripheral enhancement [122, 123]. ST-RADS III lesions (such as lipoblastomas, angio- or myolipomas, aggressive forms of fibromatosis, myositis ossificans, tenosynovial giant cell tumor) have a ≤ 2% likelihood of malignancy, while for ST-RADS IV lesions (e.g., atypical lipomatous tumor or well-differentiated liposarcoma) the risk of malignancy is 2–50% [124]. Finally, the risk of malignancy of ST-RADS V lesions is ≥ 50%, and this category includes MRI features of sarcomas (inhomogeneous solid mass, adipocytic lesion with enhancing solid nodules or thick septations, myxoid changes, T2-hyperintense enhancing mass) (Supplementary Fig. 4) [21, 125]. Biopsy is strongly advised for this last category.

Teaching point: ST-RADS categorizes soft tissue masses on MRI (0–VI) based on morphology and enhancement features, guiding suspicion of malignancy and the need for biopsy.

## TI-RADS

The American College of Radiology Thyroid Imaging Reporting and Data System (ACR-TIRADS), published in 2017, is a point-based, ultrasound classification designed to standardize the interpretation and reporting of incidental thyroid nodules to avoid unnecessary fine-needle aspirations (FNAs) [126].

**Fig. 11:**
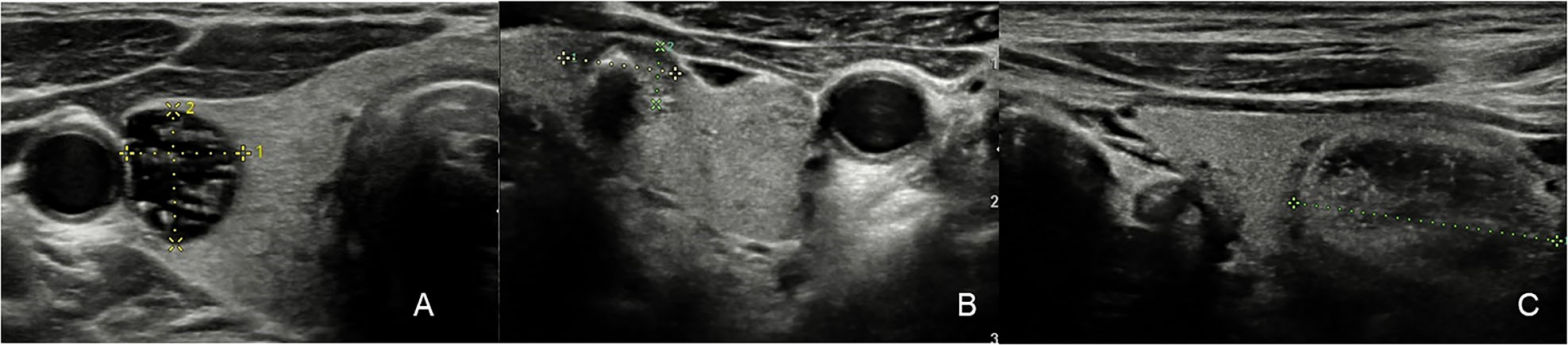
Examples of internal echogenic foci: **A** anechoic with large comet-tail artifacts (0 points) in a TR-2 nodule; **B** macro-calcifications hiding internal ecostructure (1 point) in a TR-4 nodule; **C** mixed cystic and solid, isoechoic, with punctate echogenic foci (3 points) in a TR-4 nodule. The latter are smaller than macro-calcifications and non-shadowing. Moreover, in contrast to large comet-tail artifacts, they may present the same artifacts but less than 1 mm in depth

Nodules are classified as benign, not suspicious, minimally suspicious, moderately suspicious and highly suspicious for malignancy through five relevant sonography features: composition, echogenicity, shape, margin and echogenic foci [126]; each characteristic has a score, and the sum determines the risk category. Recommendations for FNA or follow-up depend on risk category and maximum diameter [126]. No more than four nodules for follow-up and two nodules for FNA should be reported [126]. Features of benignity are cystic or almost completely cystic and spongiform compositions in which follow-up is not required; notably, a nodule is considered spongiform only if more than half of its content consists of small cystic spaces [127]. For mixed cystic and solid nodules, suspicious features are an acute angle between the eccentric solid part and the nodule’s wall, and the presence of flow on color Doppler [126]. Among the suspect features, those that carry the most points are high hypo-echogenic appearance, evaluated relatively to strap muscle, the taller than wide shape (assessed on axial plane), and extrathyroidal extension, which should be suspected if the characteristics of the nodule are generically malignant [128]. When assessing internal echogenic foci, differentiating large comet-tail artifacts, macro-calcifications and punctate echogenic foci may be difficult (Fig. 11).

TI-RADS has shown the highest sensitivity among all the proposed reporting systems [129–132]. A 2021 meta-analysis reported pooled sensitivity and specificity of 89% and 70%, respectively [133]. The main limit is interobserver variability, with a recent study reporting values of 50% and 79% respectively, for TI-RADS levels and FNA recommendations [134].

Like ACR TI-RADS, EU (European) TI-RADS, has also been updated in 2017 and reached a similar performance, however, showing differences in classification: EU TI-RADS 1 is given when no nodule is found, EU TI-RADS 2 means benign nodule, cystic or spongiform, EU TI-RADS 3 and 4 (in order, low and intermediate risk) are given one the base of echogenicity, respectively hyper- or hypo-echogenic, lastly, if at least 1 high-risk features is present (non-oval shape, irregular margins, microcalcifications, and marked hypoechogenicity) the nodule is considered high risk (EU TI-RADS 5) [135].

Teaching point: TI-RADS assigns thyroid nodules into risk-based categories using ultrasound features and a point system to reduce unnecessary FNAs while maintaining diagnostic sensitivity.

## VI-RADS

The Vesical Imaging Reporting and Data System (VI-RADS) was developed in 2018 by a panel of experts as a scoring method based on multiparametric MRI imaging [136, 137]. The staging of bladder cancer is based on the invasion of the bladder muscle, and it can be divided into non-muscle-invasive bladder cancer, confined to the urothelium and lamina propria, and muscle-invasive bladder cancer, invading the detrusor muscle with a strong association with metastasis [138].

VI-RADS proposes an MRI protocol for bladder imaging, including T2-weighted, DWI, and DCE-MRI sequences. For T2-weighted sequences, at least two planes (axial, coronal and sagittal) without fat suppression are obtained [136]. The DCE-MRI protocol provides an initial-contrast image (midline of k-space is filled) at 30 seconds after the beginning of injection and followed by the same sequences four to six times every 30 seconds to reveal the early enhancement of the inner layer, followed by tumor enhancement [136].

**Fig. 12:**
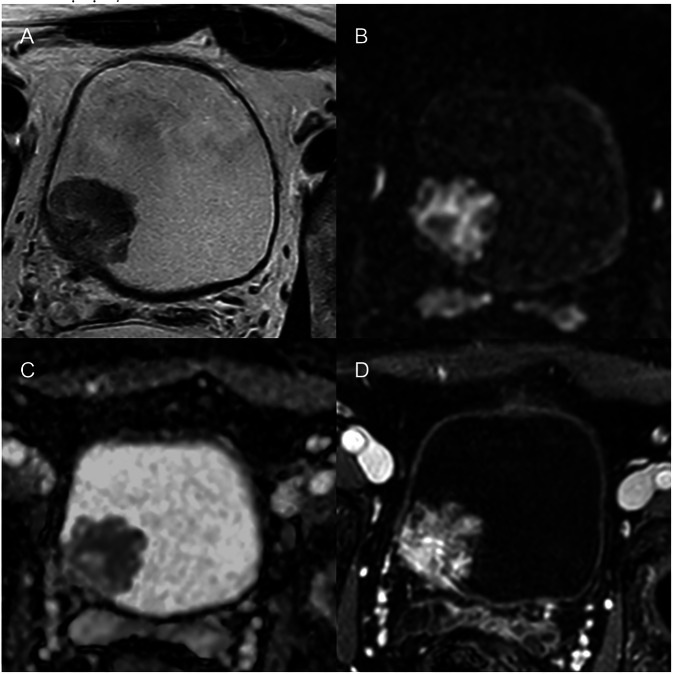
MRI in a 57-year-old male with bladder cancer. T2-weighted (**A**), DWI (**B**), ADC map (**C**), and post-contrast (**D**) images show a 3 cm lesion in the right posterior wall of the bladder with infiltration of the muscularis propria, consistent with VI-RADS 4

A 5-point assessment scale for each sequence obtains an overall VI-RADS score consisting of five categories, where scores 1 and 2 are assigned to tumors unlikely to invade the muscularis propria, scores 4 (Fig. 12) and 5 are assigned to bladder cancer likely to infiltrate the detrusor muscle layer, and score 3 represents an indeterminate or equivocal case [136]. The VI-RADS categories were associated with high sensitivity and specificity for the discrimination between non-muscle-invasive and muscle-invasive BC in recent studies [139].

Teaching point: VI-RADS scores bladder tumors on MRI (1–5) based on depth of invasion signs across T2W, DWI, and DCE, aiding preoperative staging and differentiating muscle-invasive disease.

## Conclusion

RADS are expected to be widely used in clinical practice in the near future. Therefore, radiologists need to familiarize themselves with the specific algorithms and imaging features to improve the reporting and management of patients. RADS remain dynamic documents, constantly evolving over the years according to the data provided in the literature and to the new advances in diagnostic imaging. Future improvements should consider the integration of clinical management and recommendations for treatment according to the RADS categories, as some systems remain only descriptive. It is expected that RADS will be progressively endorsed by clinical practice guidelines worldwide, and other RADS are in development for specific pathologies.

## Supplementary information

ELECTRONIC SUPPLEMENTARY MATERIAL

## Data Availability

No new data were generated or analyzed in this study. Data sharing is not applicable to this article.

## Abbreviations

ADC: Apparent diffusion coefficient
CAD: Coronary artery disease
CEM: Contrast-enhanced mammography
CEUS: Contrast-enhanced ultrasound
CT: Computed tomography
CTC: Computed tomography-colonography
CT-FFR: Computed tomography fractional-flow-reserve
DCE: Dynamic contrast-enhanced
DWI: Diffusion-weighted imaging
FNA: Fine-needle aspiration
HCC: Hepatocellular carcinoma
MRI: Magnetic resonance imaging
PET-CT: Positron emission computed tomography
RAC: Response assessment categories
RADS: Reporting and Data System
US: Ultrasound
WB-MRI: Whole-body MRI
