# Supplementary material for: RADS ALPHABET: news and tips for young and general radiologists
Source: Insights Imaging. 2026 Jan 12;17:9. doi: 10.1186/s13244-025-02154-8 (PMC12796044; doi:10.1186/s13244-025-02154-8)
Supplement: Supplementary file 1 — ELECTRONIC SUPPLEMENTARY MATERIAL [file 13244_2025_2154_MOESM1_ESM.pdf]

RADS ALPHABET: news and tips for young and general radiologists

ELECTRONIC SUPPLEMENTARY MATERIAL

Insights Imaging (2025) Cannella R, Lanza C, Pellegrino G, et al.

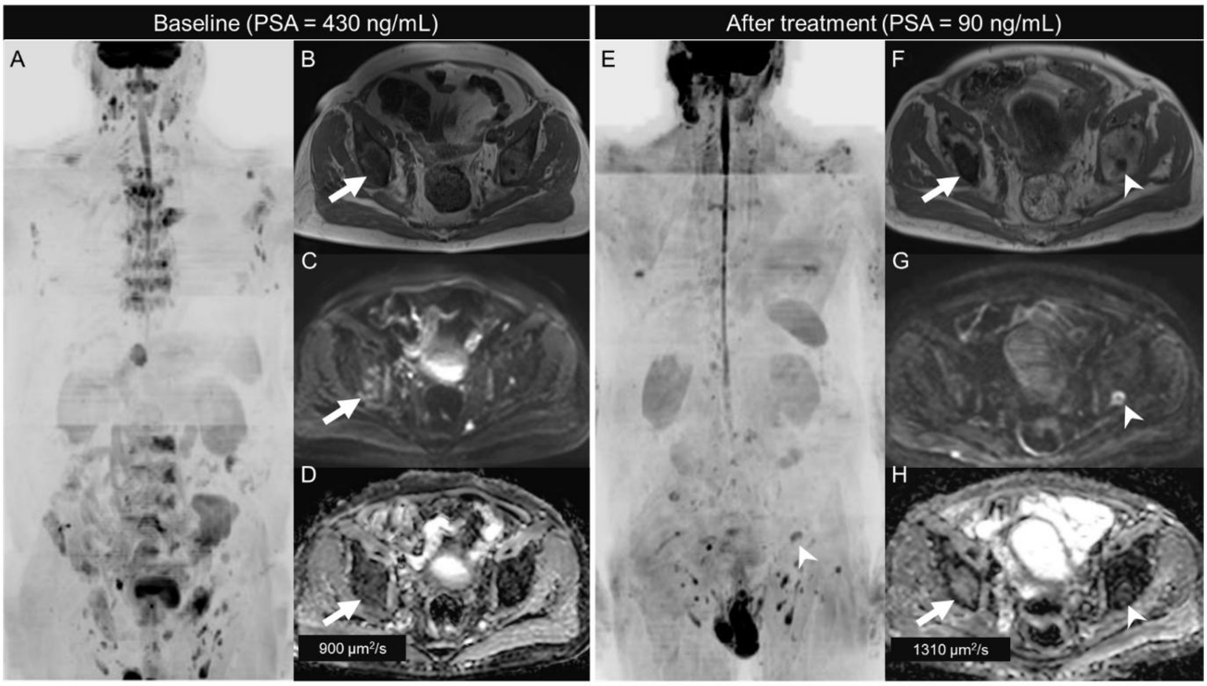

Supplementary figure 1: Whole-body MRI performed according to MET-RADS-P guidelines in a patient with metastatic castration resistant prostate cancer, before (left) and after treatment with cabazitaxel (right). In the coronal maximum intensity projection of high b-value DWI, displayed in inverted grayscale (A), multiple metastases with high signal intensity are visible in the spine, pelvis and thorax. Axial sections of the pelvis are shown, including T1-weighted images (B), high b-value DWI (C), and ADC map (D), depicting a large metastasis in the right iliac bone (arrows). After treatment, marked reduction in DWI signal intensity of most metastases can be observed (E), with concomitant increase in ADC values (+46%) (arrow in H), consistent with response assessment category 1 (RAC 1, highly likely to be responding). However, there is also an increase in size of a metastasis in the left iliac bone (arrowheads in E, F, G, H), with high signal intensity in DWI and low ADC value, consistent with RAC 5 (highly likely to be progressing). This is a case of heterogeneous response, with primary RAC 1 and secondary RAC 5 patterns.

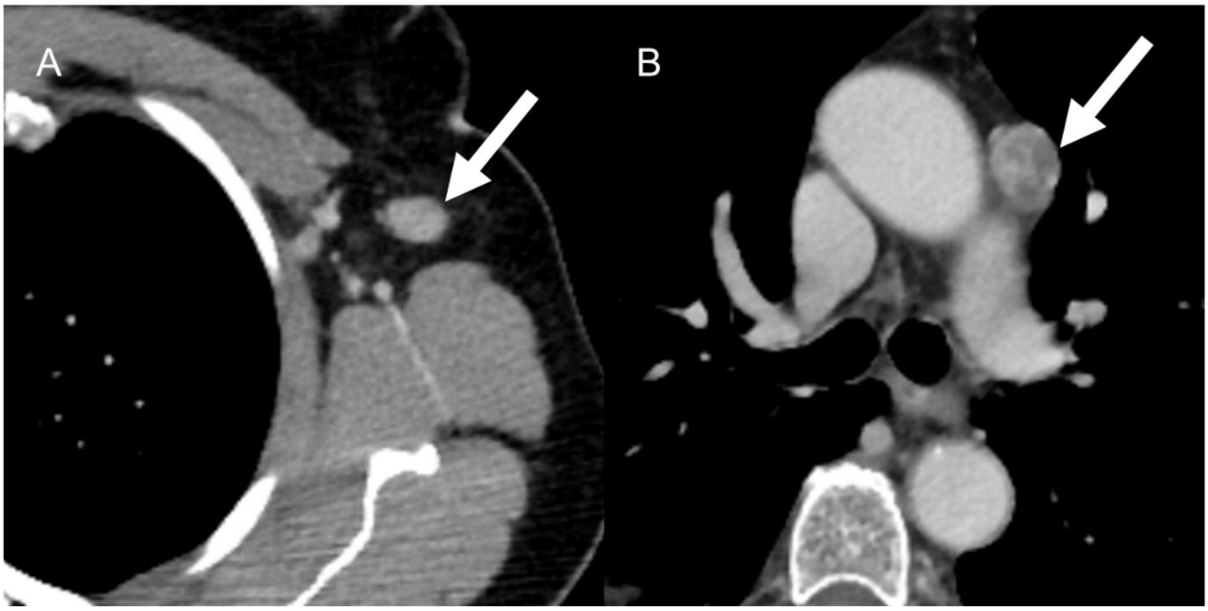

Supplementary figure 2: (A) Example of Node-RADS 2: enlarged (short axis >10 mm, but no bulk) axillary lymph node (arrow in A), with homogeneous texture, smooth border, and kidney-bean-like shape. (B) Example of Node-RADS 5: enlarged paraaortic lymph node (arrow in B), with necrosis, smooth border and spherical shape without fatty hilum.

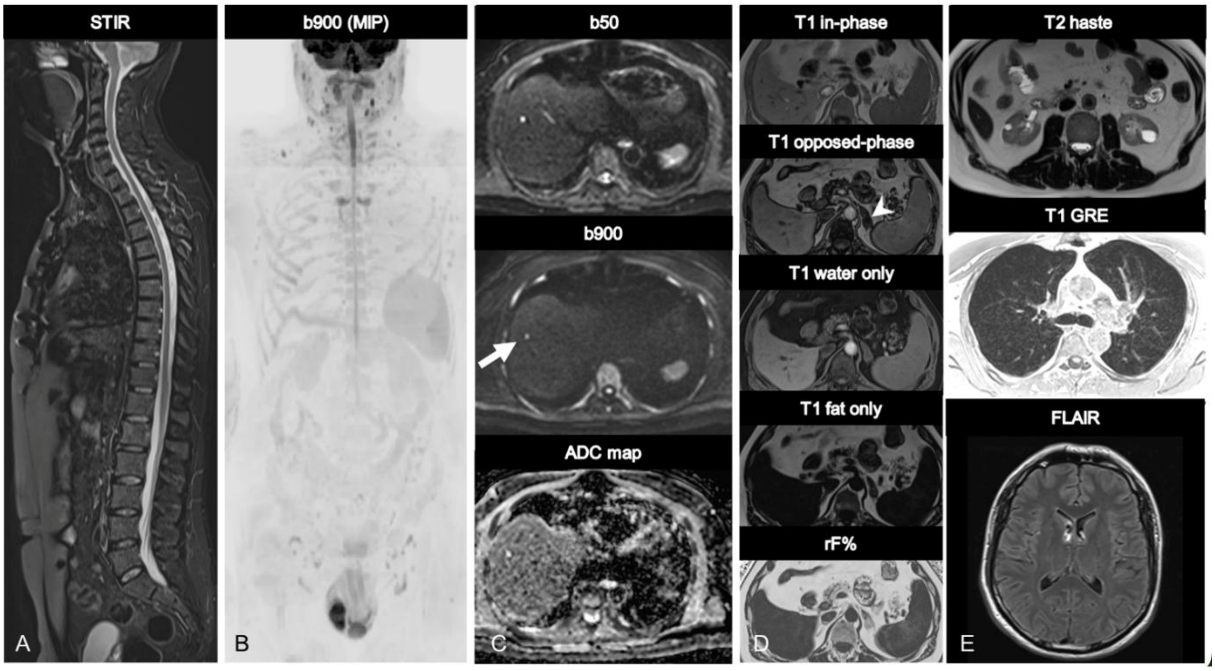

Supplementary figure 3: An approximately 60-year-old patient imaged with ONCO-RADS protocol. (A) Sagittal short inversion time inversion-recovery (STIR) T2-weighted turbo spin-echo image of the spine. (B) Coronal maximum intensity projection reconstruction of high b-value DWI displayed using an inverted gray scale. (C) Axial DWI obtained with b-values of 50 sec/mm2 and 900 sec/mm2 and corresponding ADC map. A small lesion in the right hepatic lobe, with T2 shine-through on DWI, suggestive of a hepatic hemangioma. (D) In-phase, opposed-phase, fat-only, and water-only images from axial T1-weighted gradient-recalled echo Dixon technique and relative fat fraction map. Left adrenal lesion with intracellular fat content, indicative of adrenal adenoma (arrowhead). (E) Axial T2-weighted turbo spin-echo (TSE) image at the level of the upper abdomen with bilateral renal cysts, T1-weighted image of the lung, and T2-weighted fluid-attenuated inversion recovery (FLAIR) image of the brain.

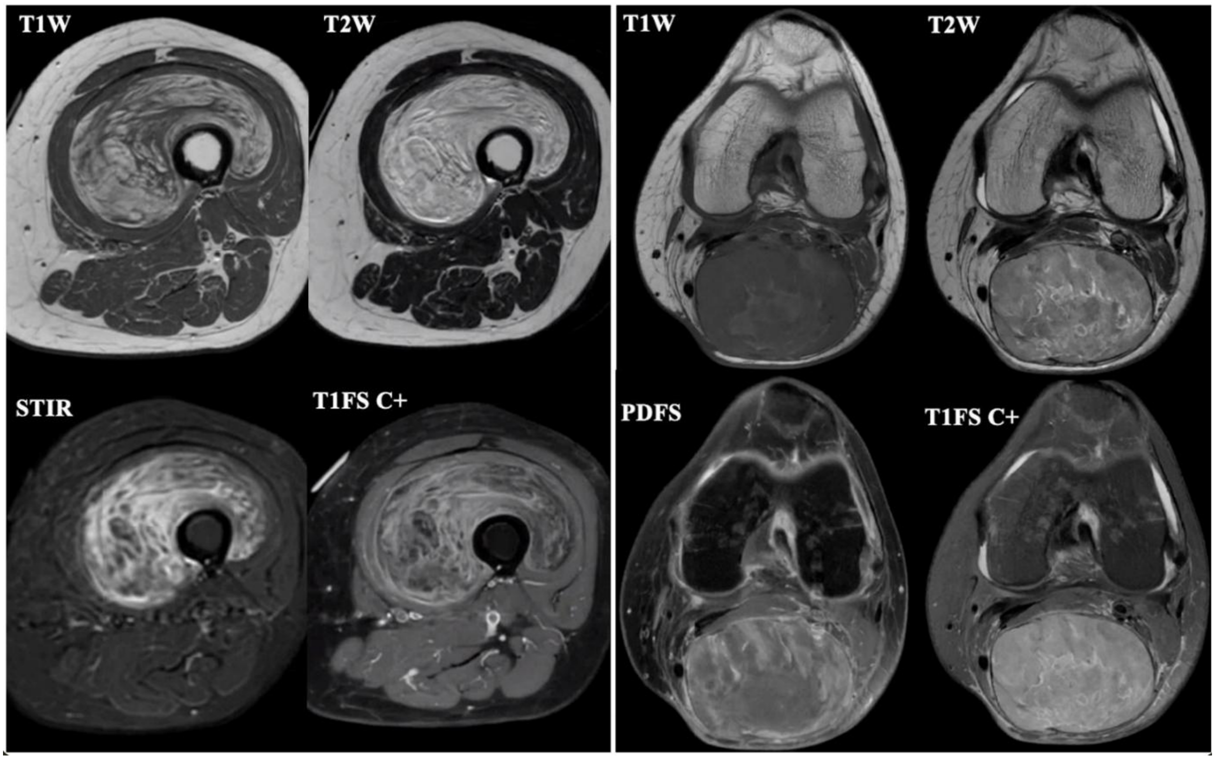

Supplementary figure 4: Two images of ST tumors, showing an atypical lipomatous tumor of the thigh (left side) and a high-grade sarcoma of the popliteal fossa (right side). The different sequences clearly show the higher but inhomogeneous adipose content of lipomatous lesion with multiple enhancing septations, without clear myxoid degeneration or solid focal nodule. On the contrary, the sarcoma on the right presents as a solid mixed-intensity, lipid-poor and T2 hyperintense enhancing.
